# Design and Experimental Validation of a Biomimetic Jellyfish Robot with Asymmetric-Stiffness Swimming Arms and Dual-Power Cooperative Drive

**DOI:** 10.3390/biomimetics11100745

**Published:** 2026-10-16

**Authors:** Zhiyuan Hu, Fulin Du, Dingsheng Feng, Qiuxuan Wu, Xiaoqing Tian, Jian Wang

**Affiliations:** 1 School of Automation, Hangzhou Dianzi University, Hangzhou 310018, China; 2 HDU-ITMO Joint Institute, Hangzhou Dianzi University, Hangzhou 310018, China; 3 School of Mechanical Engineering, Hangzhou Dianzi University, Hangzhou 310018, China

**Keywords:** biomimetic jellyfish robot, asymmetric-stiffness swimming arms, motor–spring cooperative actuation, CFD analysis, experimental evaluation

## Abstract

This study presents a biomimetic jellyfish robot integrating asymmetric-stiffness swimming arms with motor–spring cooperative actuation. Motor-driven spring charging and rapid spring release organize the recovery, power-stroke, and gliding phases. Hinged arm segments, return micro-springs, and a limiting structure are designed to permit recovery-stroke bending while resisting bending during the power stroke. Component CFD and MATLAB(R2024a) motion-cycle analysis provide preliminary hydrodynamic and structural configuration comparisons. An arithmetic check of the retained drag fits gives a velocity-dependent arm-drag reduction of approximately 52.87–54.41% over 0.10–0.30 m/s and a separate body-drag reduction of approximately 44.49%; these are fitted-model comparisons, not measured drag reductions or mesh-independent predictions. In the recorded water-tank experiments, the prototype traveled approximately 1.8 m over approximately 9 s, corresponding to an overall average straight-line speed of approximately 20 cm/s. The average speed during the combined power-stroke and gliding phases was approximately 27 cm/s, and the maximum turning angular velocity was approximately 4.2°/s. These observations support the engineering feasibility of the integrated prototype under the reported test conditions. Quantitative stiffness characterization, independent trial variability, and matched-timing model validation are not established from the retained records.

## 1. Introduction

Marine environmental monitoring, resource exploration, and special underwater operations continue to drive the development of underwater robots toward low disturbance, low noise, and high maneuverability [1,2,3]. Compared with propeller-driven systems, biomimetic underwater robots can better exploit biological locomotion principles and achieve smoother and more compliant propulsion and maneuvering in complex environments [3,4]. Among various biological models, jellyfish rely on the periodic contraction and expansion of their bells to realize jet propulsion, which features a simple structure, smooth propulsion, and strong environmental adaptability. Therefore, jellyfish have become an important biological prototype in underwater biomimetic propulsion research [5,6,7,8,9].

Jellyfish-inspired robots have been developed using IPMC actuation [5], motor-driven mechanisms [6,10,11,12], SMA actuation [13,14,15,16], and soft fluidic or origami structures [7,8,9]. These approaches offer different combinations of structural compliance, actuation timing, and propulsion geometry. The present study focuses on integrating motor-driven elastic energy storage with a swimming-arm mechanism intended to reduce the flow-facing area during recovery and retain arm shape during the power stroke. This integration is evaluated at the component model and prototype levels; it does not introduce a new biological propulsion principle.

The recovery, power-stroke, and gliding phases provide a framework for assigning different mechanical functions within a locomotion cycle. Translating this framework into the robot involves two design questions: how to organize slow motor-driven energy storage and rapid spring release, and how to permit direction-dependent bending of the swimming arms. The proposed design combines these functions within one prototype, allowing the actuation architecture and arm geometry to be examined together without assuming that their individual performance contributions have been isolated.

The highlights of this study are the integration of motor–spring cooperative actuation and hinged, direction-dependent swimming arms; preliminary component CFD and MATLAB configuration comparisons; and recorded straight-swimming and turning observations of the prototype. The water-tank summaries report approximately 1.8 m travel over approximately 9 s, an overall average speed of approximately 20 cm/s, approximately 27 cm/s averaged during the combined power-stroke and gliding phases, and a maximum turning angular velocity of approximately 4.2°/s. The contribution is a mechanism–structure–function integration evaluated for engineering feasibility; numerical drag comparisons, mechanical stiffness, and the isolated actuation benefit are not presented as quantitatively validated experimental results.

**Figure 1:**
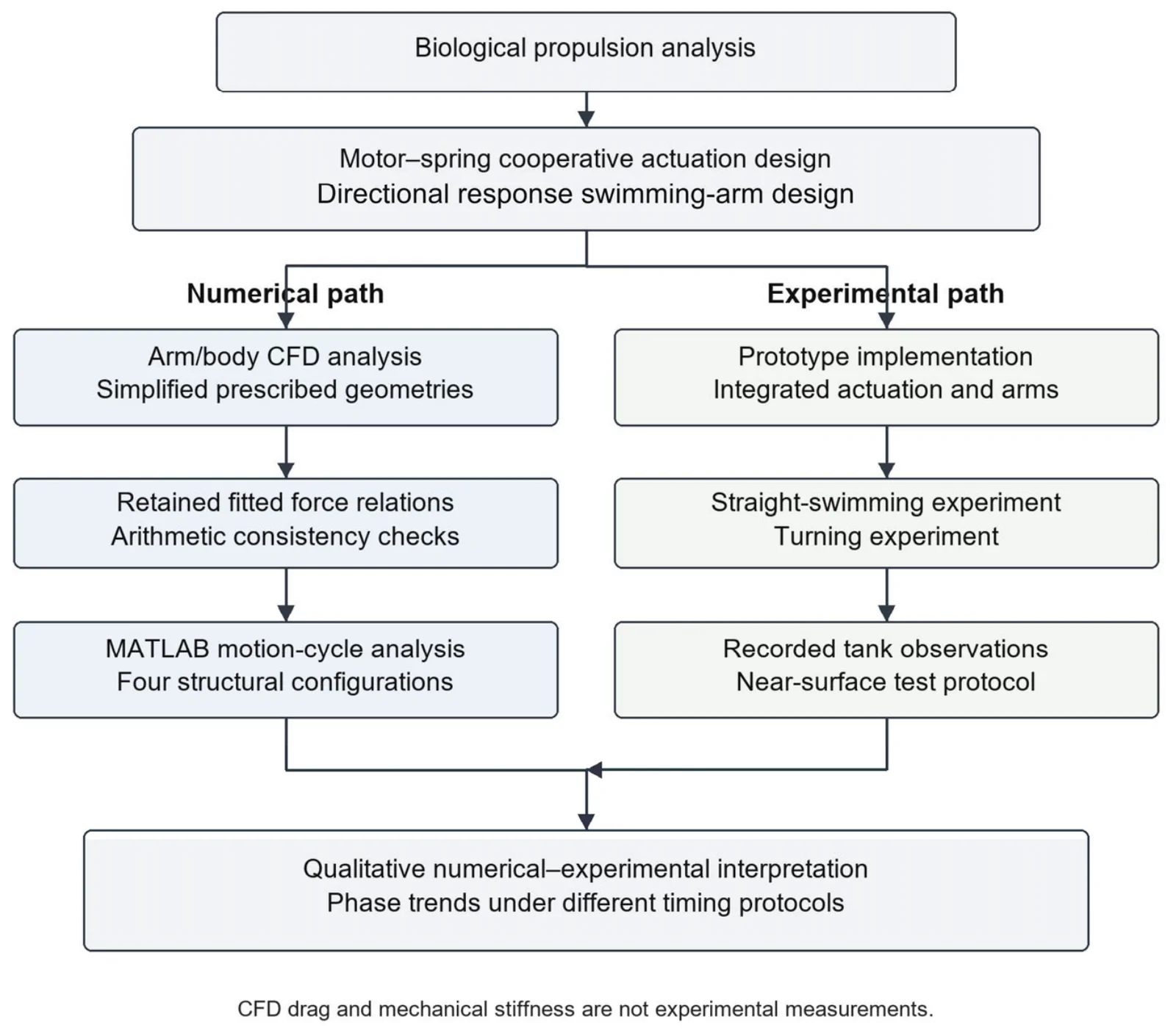
Research workflow based on the retained design, CFD, MATLAB, and prototype records. The branches meet at qualitative interpretation under different timing protocols; no coupled FSI or matched quantitative validation is implied.

Figure 1 summarizes the research steps and distinguishes the numerical and experimental evidence paths. The source design, fitted relations, and test records are described in [17].

## 2. Biomimetic Design Principle and Robot Architecture

### 2.1. Biomimetic Propulsion Principle

The core of biomimetic jellyfish propulsion does not lie merely in morphological imitation but in the organization of mechanical functions in different phases. The recovery stroke mainly serves shape recovery and drag reduction. The power stroke mainly generates propulsion through water displacement. A short gliding phase then follows to maintain forward velocity [5,6,7]. Based on this understanding, the biological inspiration is abstracted into two engineering principles. At the actuation level, the system should possess an output rhythm of “slow energy storage–rapid energy release”. At the propulsion-component level, the system should possess a functional switching capability of “drag reduction during the recovery stroke–thrust preservation during the power stroke.”

### 2.2. Overall Structure and System Composition of the Robot

**Figure 2:**
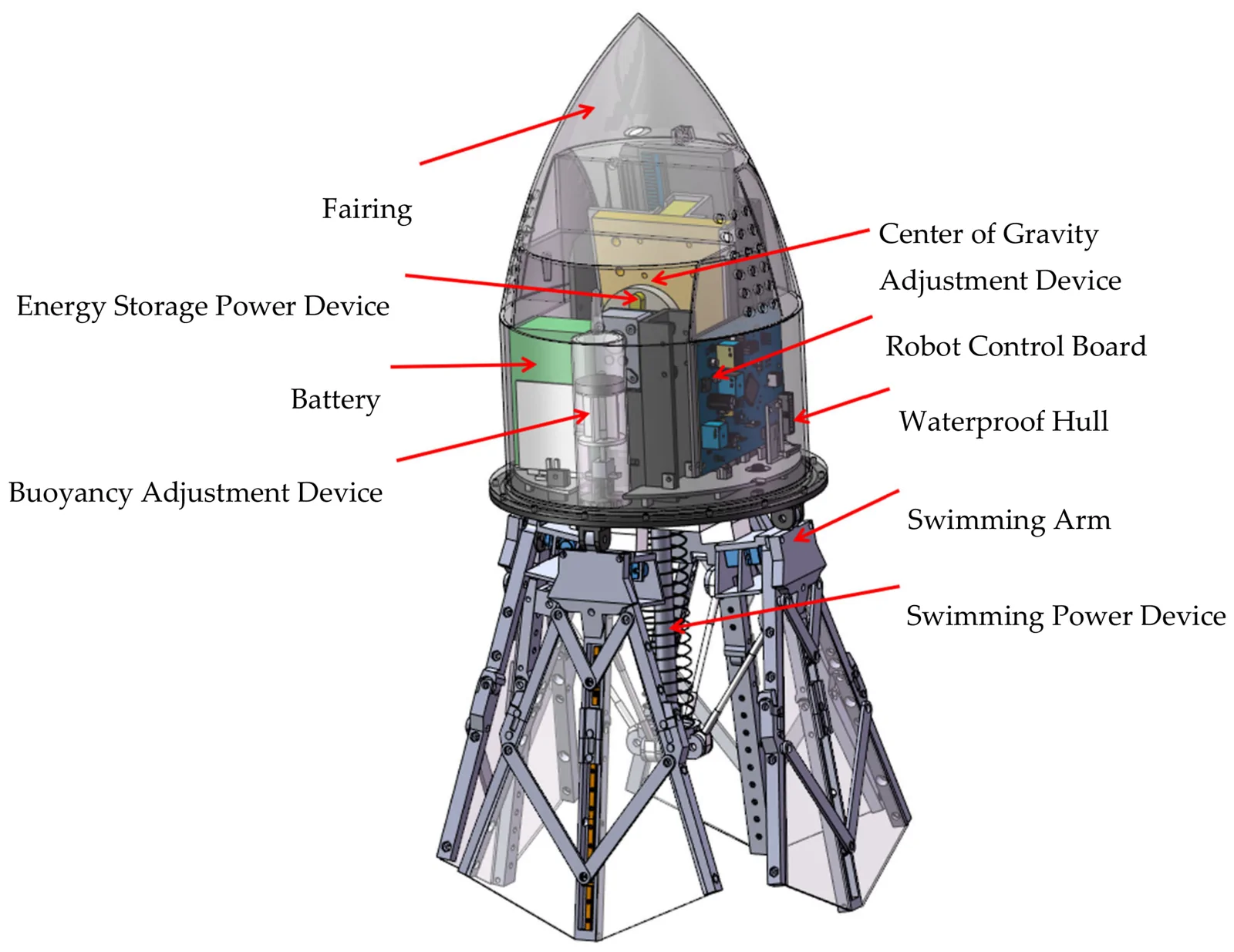
Overall robot CAD structure used for prototype fabrication. Overall dimensions: 740 mm × 270 mm × 270 mm; body diameter and length: 270 mm and 390 mm.

The biomimetic jellyfish robot consists of a head body and four swimming arms, forming an integrated “body–actuation–execution–regulation” system, as shown in Figure 2. Functionally, the system can be divided into three parts: the propulsion module, the actuation module, and the state regulation module.

The propulsion module comprises four swimming arms intended to interact with the surrounding water through phase-dependent opening, closing, and bending. The actuation module comprises an energy storage device that uses a motor to compress the power spring and a swimming actuation device that transmits the released spring energy to the arms. The state regulation module comprises the center of gravity and buoyancy adjustment devices. The head body houses the power supply, controller, sensors, and ballast components.

In terms of spatial layout, the head body integrates the power supply, control board, sensors, and counterweight modules internally while externally connecting to the actuation mechanism and swimming arms to form a compact centralized structure. This structural design satisfies propulsion performance requirements while considering sealing performance, independent operation capability, and attitude controllability [10,11]. Compared with traditional biomimetic jellyfish robots based on a single actuation method or a single flexible structure, the proposed structural design emphasizes the cooperative matching between actuation timing and structural response, providing a system-level foundation for subsequent propulsion performance optimization.

### 2.3. Dual-Power Cooperative Actuation Architecture

**Figure 3:**
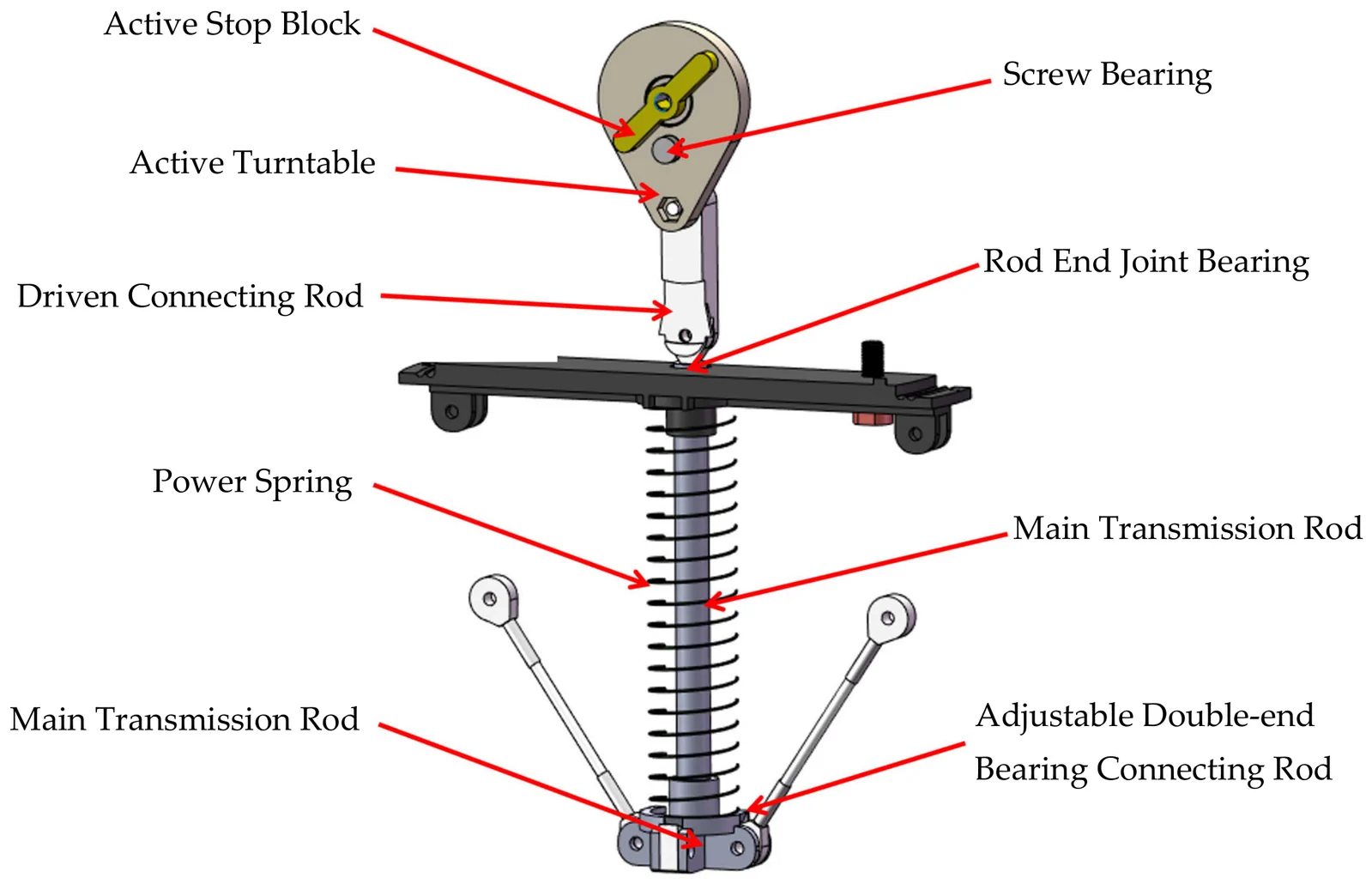
Motor–spring cooperative actuation CAD mechanism. The main transmission rod stroke is 100 mm. Power spring wire diameter, outer diameter, and free length are 2 mm, 40 mm, and 300 mm, respectively; these values do not describe the hinge micro-springs.

The dual-power cooperative actuation architecture comprises a swimming actuation device and an energy storage actuation device, as shown in Figure 3. During the recovery stroke, the motor drives the energy storage actuation device to compress the power spring. After the release position is reached, the power spring releases its stored elastic energy through the swimming actuation device, driving the swimming arms through the power stroke. The spring force decreases as the spring returns toward its initial state, while the effective thrust component also changes with the swimming-arm angle. This relationship provides the mechanical basis for matching the energy release process to the power-stroke motion.

The present results do not isolate the contribution of spring-assisted energy release relative to traditional single-motor actuation. A controlled comparison under identical operating conditions is needed to quantify this contribution.

### 2.4. Design of the Asymmetric-Stiffness Swimming Arm

The swimming arm connects the actuation mechanism to the surrounding fluid. Its design aims to combine reduced flow-facing area during recovery with resistance to bending during the power stroke. Rather than assigning a measured stiffness ratio, the term asymmetric stiffness describes this intended direction-dependent mechanical response.

**Figure 4:**
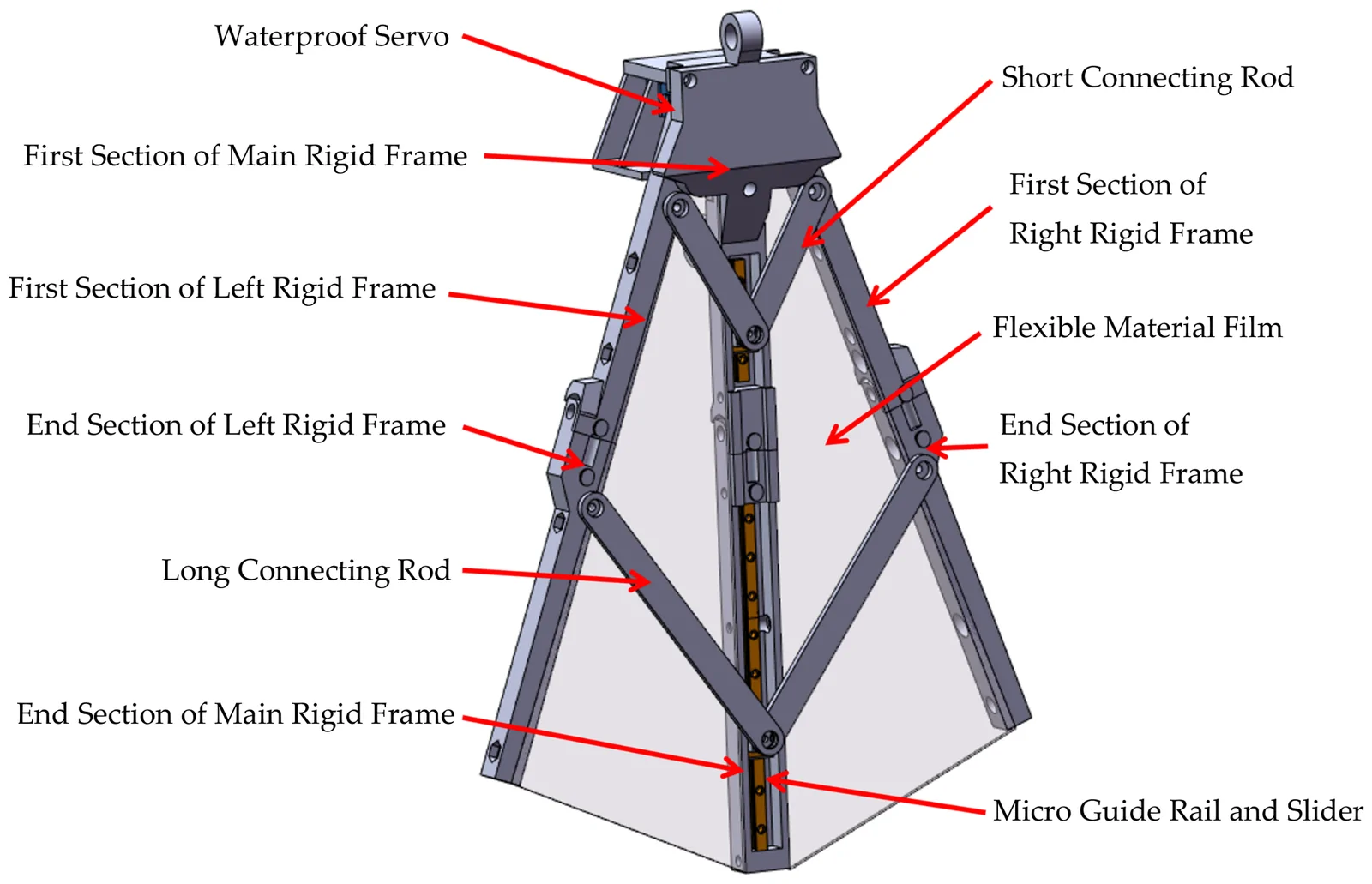
Swimming-arm CAD structure used for prototype fabrication. Arm length: 330 mm. Hinged segments, return micro-springs, and the directional stop implement the intended asymmetric response; no quantitative stiffness measurement is shown.

The proposed arm is shown in Figure 4. It includes a main rigid skeleton, left and right rigid skeletons, a flexible membrane, gears, synchronous connecting rods, return micro-springs, and a limiting structure.

The swimming arm consists of a main rigid skeleton, left and right rigid skeletons, and a flexible membrane. Each rigid skeleton is divided into two hinged segments, giving six rigid skeleton segments in a single swimming arm. The rigid skeleton components are fabricated from resin by 3D printing and assembled using bolts and nuts. The flexible membrane is fixed between the rigid skeletons. When the swimming-arm surface closes, the flexible membrane folds between the rigid skeletons; when the surface opens, the membrane is extended to increase the flow-facing area.

The membrane material and thickness, resin grade and quantitative material properties, and hinge micro-spring dimensions and stiffness are not specified in the retained fabrication records. The hinge micro-springs provide local arm return and are distinct from the actuation power spring. Table 1 reports only the documented dimensions and force parameters of the power spring. The spring-constant entry in the source has an inconsistent unit and is not used as a verified stiffness value.

During recovery, the distal hinged segments are designed to bend under fluid loading while stretching the return micro-springs. The springs assist return toward the straight configuration, whereas the limiting structure restricts bending in the power-stroke direction. The retained design provides three spring-mounting slots per arm and allows the local response to be adjusted through micro-spring length, number, and stiffness. These are design features; the present work does not quantify their separate effects on propulsion.

Measured bending stiffness, moment–angle relationships, and load–deformation curves are not available in the retained mechanical records. The magnitude of the stiffness asymmetry has therefore not been quantitatively verified experimentally. The CFD comparison concerns simplified arm geometries and does not constitute mechanical stiffness measurement.

The arm concept uses hinged segments and a directional stop to associate recovery-stroke compliance with power-stroke shape retention. Its contribution is the integration of this directional response structure with the motor–spring timing architecture. The flow-field analysis provides a hydrodynamic rationale for examining the corresponding arm configurations, while the prototype tests evaluate the integrated system rather than the arm mechanism in isolation.

### 2.5. Control System Composition

**Figure 5:**
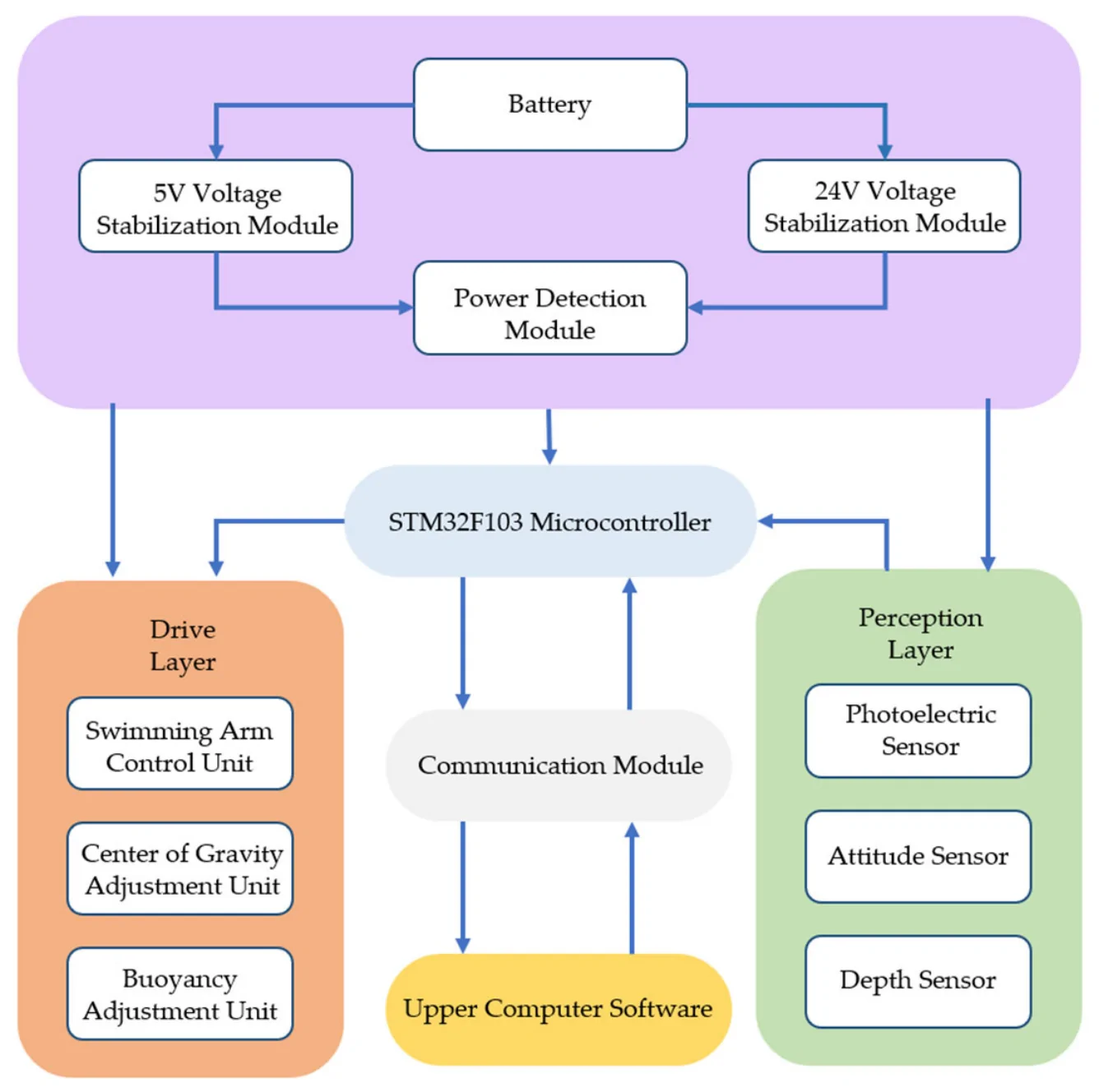
Retained control system architecture diagram. Its controller model label differs from the source textual description (Section 2.5). This diagram does not depict the overall research workflow.

The control system can be divided into three parts: the execution layer, the power supply and sensing layer, and the timing-control layer, as shown in Figure 5. The execution layer includes the swimming actuation device, energy storage actuation device, swimming-arm opening-and-closing mechanism, and unilateral closing mechanism for turning. The power supply and sensing layer includes the battery, control board, and necessary sensors. The timing-control layer organizes the action sequence of “slow recovery stroke–rapid power stroke–gliding phase”. During turning, one side of the swimming arms remains closed to generate asymmetric thrust, thereby realizing directional control.

The onboard control system coordinates four servomotors for arm-surface opening and closing and three motors for energy storage, center of gravity adjustment, and buoyancy adjustment. Depth and attitude sensors communicate through an IIC interface, and an ATK-LORA-01 module at 433 MHz provides robot–host communication. An optical encoder records main motor position and revolutions; relays and an external switch permit power disconnection. The retained textual record identifies STM32F4, whereas the source control diagram labels STM32F103. The installed controller model is therefore not confirmed by the available records; the reported architecture and functions are described without selecting between those model labels.

The prototype is independently powered by an onboard 24 V lithium battery with a capacity of 6000 mAh.

## 3. Materials and Methods

### 3.1. Prototype Implementation and Key Parameters

CFD simulations, motion-cycle simulations, and water-tank experiments were conducted to evaluate the hydrodynamic characteristics and swimming performance of the biomimetic jellyfish robot. Computational models of the swimming arm and body were established in ANSYS (2023 R1) Fluent under different flow and motion conditions. At the whole-robot level, MATLAB was used to analyze the displacement and velocity variations over successive motion cycles, each consisting of the recovery stroke, power stroke, and gliding phase. Straight-line swimming and turning experiments were then carried out to evaluate the propulsion performance and maneuverability of the prototype.

The drag fits were obtained from CFD, while the water-tank records summarize displacement, swimming speed, and turning angular velocity. The underlying design, fitted hydrodynamic relations, and prototype records are retained in the associated dissertation [18]. No direct experimental drag measurement is reported.

The prototype was implemented according to the structure in Section 2, with a head body, four swimming arms, the motor–spring actuation mechanism, and the center of gravity and buoyancy adjustment devices. Resin-printed rigid skeletons and attached flexible membranes form the arm assemblies. The prototype implements the designed energy storage and release sequence, enabling swimming and turning observations under the reported tank conditions.

Considering that the dimensions, mass, power supply, and actuation component parameters directly affect subsequent simulation and experimental results, the main physical parameters of the prototype are summarized in Table 1 and are not repeated in the main text.

### 3.2. Timing and Control Method of Dual-Power Cooperative Actuation

The core of dual-power cooperative actuation lies in the reasonable organization of actuation timing. In this study, a single motion cycle of the robot is divided into the recovery-stroke, power-stroke, and gliding phases. The coordinated action of the motor and elastic element is controlled to realize different power outputs in the different phases.

During recovery, motor-driven linkage motion opens the arms slowly while compressing the power spring. At the release position, the spring drives the power stroke through the swimming actuation device. The subsequent gliding phase is treated as inertial forward motion without active stroke input. Arm-surface opening and closing are coordinated with these phases through the servomotors.

The actuation cycle can be expressed as:

T = t_1_ + t_2_ + t_3_. (1)

where T is the duration of a complete motion cycle and t_1_, t_2_, and t_3_ are the durations of the recovery stroke, power stroke, and gliding phase, respectively.

In the water-tank experiments, the duration of the recovery stroke was set to 1.2 s and that of the gliding phase was set to 1.0 s. The total duration of a single motion cycle was approximately 3 s. The energy storage frequency and gliding duration could be adjusted through the host computer interface.

The phase durations specify the actuation protocol. They are reported here to distinguish the experimental timing from the MATLAB motion-cycle timing and are not treated as a quantitatively optimized control schedule.

The motor–spring arrangement provides a mechanical implementation of slow energy storage and rapid release. This describes the actuation sequence; a performance advantage over motor-only actuation requires a controlled comparison that is not available in the retained records.

### 3.3. CFD Simulation Setup

Component CFD models were established in ANSYS Fluent for the power stroke, rigid-arm recovery, asymmetric-arm recovery, and body flow field. The retained setup uses an incompressible water representation and the k–ε model family. The analyses provide preliminary hydrodynamic comparisons under the recorded assumptions.

For the arm analysis, a simplified prescribed geometry was imported from the geometric model. A compiled UDF imposed arm rotation, and the dynamic mesh accommodated this prescribed motion; inlet flow represented relative robot translation. The asymmetric-arm recovery case is interpreted as a comparison using an imported simplified bent configuration. A structural solver or coupled FSI calculation is not documented. Thus, the CFD results are not interpreted as FSI-predicted, load-dependent arm deformation. The exact bent profile and the procedure used to create it cannot be recovered from the retained records.

The Reynolds number is defined by Equation (2), where ρ and μ denote water density and dynamic viscosity, U is inlet velocity, and L is characteristic model length. The retained model lengths span 0.2–0.5 m, and inlet velocities span 0.10–0.30 m/s. The water property values and temperature needed to reproduce the numerical Reynolds number range are not specified in the retained settings, so a single reconstructed range is not asserted.

Re = ρUL/μ.(2)

The retained CFD description identifies the k–ε model family and cites computational cost as part of the selection rationale [18]. The particular variant and near-wall treatment are not specified. No comparison among turbulence closures is available; therefore, the model choice is reported as part of the original setup rather than as a validated choice for all flow features.

The arm model used a rectangular fluid domain. Its inlet and side boundaries were placed two characteristic lengths from the model and its outlet five characteristic lengths away. The documented conditions are a velocity inlet, a pressure outlet, and no-slip walls. The inlet velocities were 0.10, 0.15, 0.20, 0.25, and 0.30 m/s. The body comparison instead used a cylindrical fluid domain [18]; its detailed domain dimensions and boundary specification cannot be recovered, and the arm-domain settings are not assigned to the body model.

The initial dynamic mesh region surrounding the arm was meshed more densely than the remaining domain, and Fluent mesh checks were used to examine overlapping or irregular elements. Mesh type, element count, minimum element size, quantitative refinement settings, boundary layer specification, wall treatment, and y+ are not specified in the retained records. These omissions limit reproduction of the original mesh.

The associated dissertation includes a residual-versus-iteration plot for an arm steady-flow case, extending to approximately 500 iterations [18]. This image is not a recoverable residual time series or a complete convergence record. The Fluent version, solver type, k–ε variant, pressure–velocity coupling, discretization schemes, convergence thresholds, transient time step, and iterations per time step are not reliably recoverable. An iteration count alone does not establish convergence, and the residual image does not demonstrate force convergence or convergence of every CFD case.

Mesh independence is not established from the retained simulation records: drag or thrust results at multiple mesh densities are not available. Accordingly, the fitted force relations are used for preliminary model comparisons without claiming mesh-independent absolute predictions or numerical uncertainty bounds.

### 3.4. Motion-Cycle Simulation Method

MATLAB motion-cycle analysis used the linkage kinematics, single-arm rotational dynamics, and longitudinal force balance recorded in the underlying model [18]. Four arms were assumed to move synchronously and symmetrically in vertical planes. The linkage torque and hydrodynamic resistance torque determine the arm angular acceleration. At the robot level, the phase-specific force balance distinguishes recovery resistance, power-stroke thrust, and gliding resistance.

The driving spring force is transmitted through the main rod and connecting rods and is distributed equally among the four arms in the symmetric model. The retained dynamics describe a linear spring–force relation with transmission position; the numerical coefficients and a verified spring constant are not recoverable. The reported power spring parameters are a 21 N preload, 100 mm maximum compression, and 70 N maximum output force. The model uses CFD-derived arm thrust and resistance relations and the body-drag fits.

The model relationships are summarized in Equations (3)–(5). Here, I_a_ is the arm rotational inertia, τ_link_ is linkage driving torque, τ_hydro_ is hydrodynamic resistance torque, m is robot mass, F_T_ is the effective longitudinal thrust of one arm, F_D,r_ is the recovery resistance of one arm, and F_D,b_ is body resistance. Their signs follow the forward motion convention of the retained model. These equations clarify the model structure; missing input and solver records prevent a fully reproducible reconstruction of the original MATLAB calculation.

I_a_ dω/dt = τ_link_ − τ_hydro_. (3)

F_h_ = −4F_D,r_ − F_D,b_ (recovery);

F_h_ = 4F_T_ − F_D,b_ (power stroke);

F_h_ = −F_D,b_ (gliding). (4)

dv/dt = F_h_/m. (5)

Four complete motion cycles were simulated with an initial robot velocity of zero. The displacement, velocity, acceleration, and resultant force were calculated to characterize the motion of the robot throughout the swimming process.

The MATLAB analysis used approximately 5 s per cycle, including a fixed 3 s gliding phase, over four cycles. The tank protocol used approximately 3 s per cycle, including 1.2 s recovery and 1.0 s gliding. The reason for choosing different protocols is not specified in the retained records. The duration of resistance and inertial decay consequently differs between the two cases, so their absolute displacement and velocity are not compared as matched-timing validation. Interpretation is restricted to qualitative phase-dependent motion trends.

### 3.5. Experimental Setup and Measurement Method

Experiments were conducted in a 4 m × 2 m tank with 1 m water depth, using the onboard 24 V, 6000 mAh lithium battery and no external power or signal cables. For straight swimming, recovery was set to 1.2 s and gliding to 1.0 s, with a complete cycle of approximately 3 s. A recorded sequence comprised three consecutive cycles over approximately 9 s. The source describes buoyancy adjustment so that the robot floated at the water surface, with the head slightly raised from the horizontal [18]. These are near-surface tank observations and should not be generalized to fully submerged operation.

For turning, the swimming-arm surface on the side toward the turning direction remained closed, while those of the other three swimming arms opened during the power stroke. This difference in thrust generated a turning moment. After the turn was completed, all four swimming arms resumed the normal actuation sequence.

Displacement, speed, and turning angle values are reported as recorded experimental summaries. Camera resolution and frame rate, calibration scale, tracked reference point, tracking software or algorithm, pixel-to-distance conversion, and the velocity-processing procedure are not recoverable from the retained measurement records. A calibrated video metrology workflow and numerical measurement uncertainty estimate are therefore not claimed.

The source mentions multiple repetitions of the straight-swimming test [18], but does not specify the number of independent trials, resetting procedure, or individual trial results. Three consecutive cycles within one sequence are observations of that sequence, not three independent experiments. The summaries do not support an independent trial sample size, mean ± standard deviation, or error bars.

## 4. Simulation Results

### 4.1. Numerical Simulation of Robot Motion Cycle

**Figure 6:**
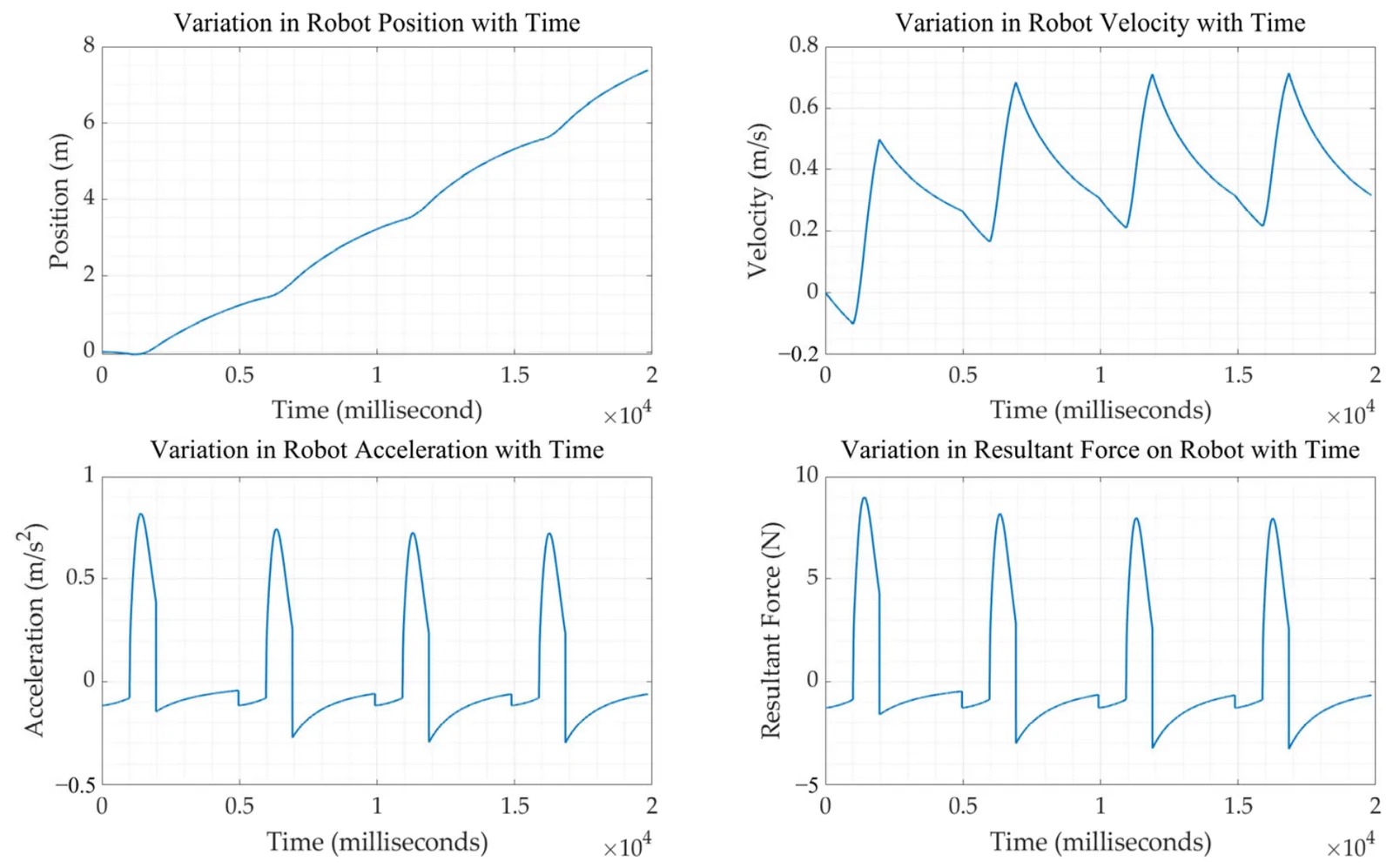
MATLAB motion-cycle results for single- and multiple-cycle analysis using approximately 5 s per cycle and a fixed 3 s gliding phase.

The MATLAB results describe phase-dependent displacement and velocity in single- and multiple-cycle motion, as shown in Figure 6. The simulated velocity decreases during recovery and may become briefly negative, rises during the power stroke, and decays during gliding. These trends are used to interpret the tank observations qualitatively under the different timing protocols, rather than to establish prediction accuracy.

### 4.2. Flow-Field Characteristics During the Power-Stroke Phase

**Figure 7:**
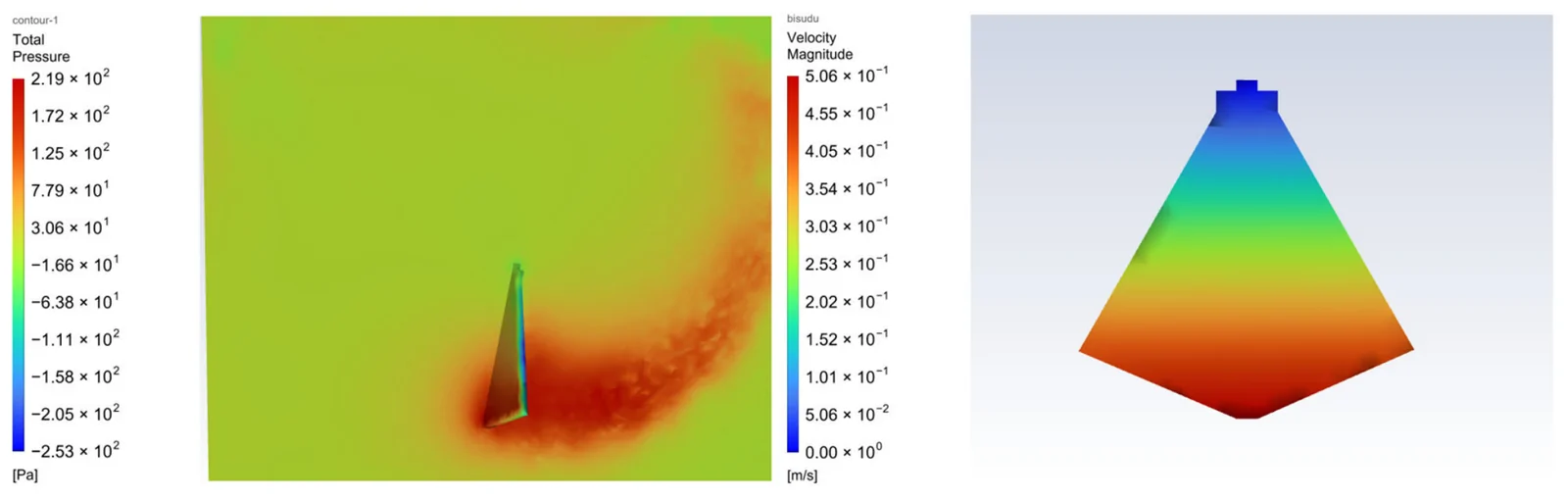
Reported pressure and velocity contours for the prescribed arm power-stroke configuration with UDF-imposed rotation and dynamic mesh. The retained record does not establish numerical uncertainty or convergence for this case.

The power-stroke pressure and velocity contours describe flow around the prescribed arm configuration, as shown in Figure 7. The reported pressure distribution and wake velocity provide a hydrodynamic rationale for retaining arm shape during water displacement. They do not measure bending stiffness or isolate an experimentally verified thrust increase caused by the directional stop.

### 4.3. Drag Comparison During the Recovery Stroke

**Figure 8:**
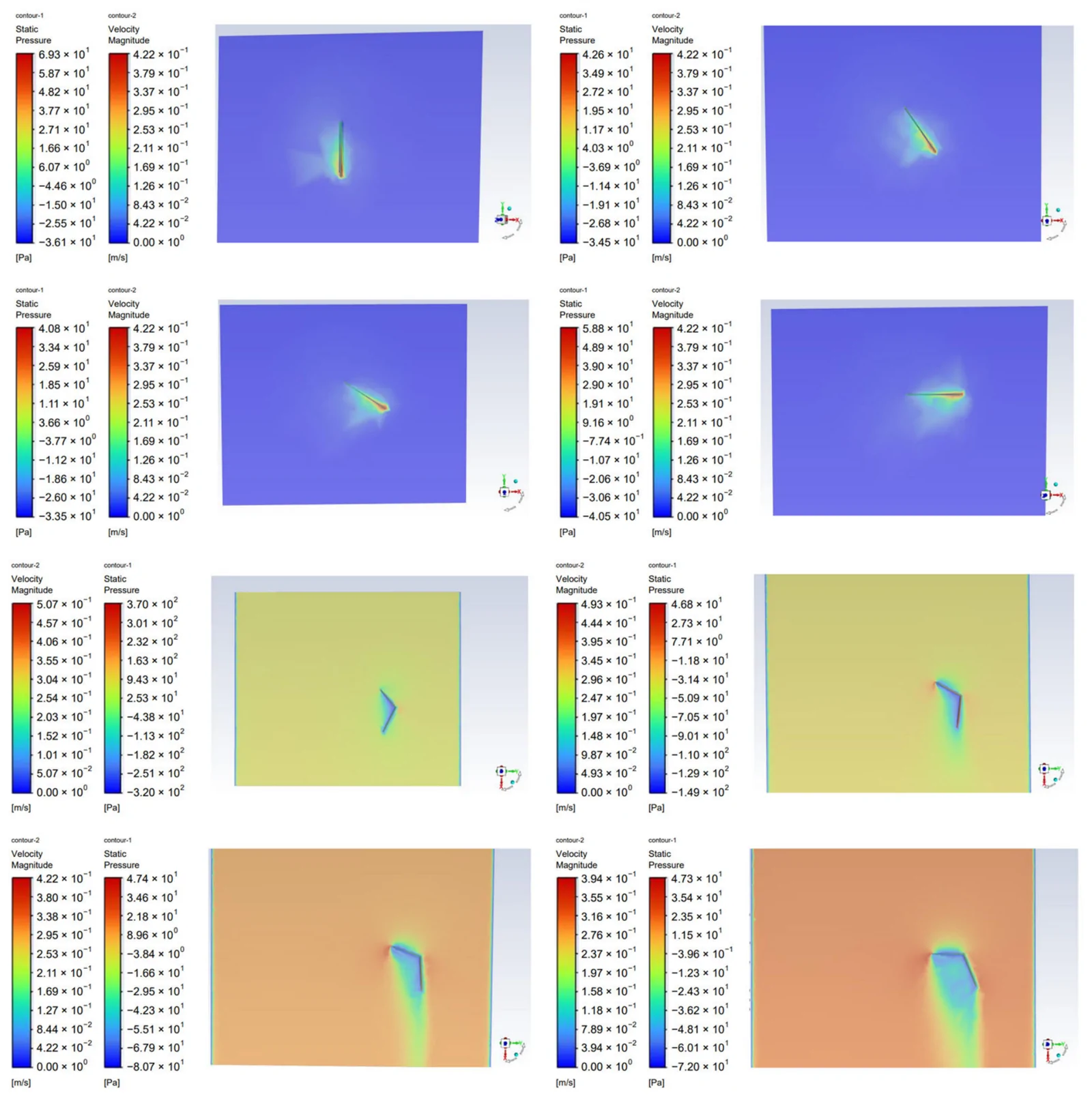
Reported recovery-stroke flow fields for rigid and simplified asymmetric-arm configurations. This is a geometric CFD comparison, not a measurement of stiffness or coupled FSI deformation.

The corresponding recovery-stroke flow fields for the rigid and simplified asymmetric-arm configurations are shown in Figure 8. The retained recovery-drag fits are given in Equations (6) and (7) [18]. The rigid-arm relation was obtained by separately fitting rotation-induced resistance and inlet-flow resistance and combining the terms; the asymmetric-arm relation was fitted for the imported simplified configuration. Inlet velocities were 0.10–0.30 m/s in steps of 0.05 m/s, and recovery rotation was described as having a fixed angular velocity. The angular velocity value and exact bent profile are not specified. Here, v is the inlet velocity in m/s and θ is the arm angle relative to the robot travel direction.

F_D,rigid_ = (0.6746 + 12.5v^2^) cosθ. (6)

F_D,asymmetric_ = (0.3214 + 5.544v^2^) cosθ. (7)

For a common angle with nonzero cosθ, the ratio in Equation (8) gives the relative difference between the fitted projected forces. At v = 0.10, 0.15, 0.20, 0.25, and 0.30 m/s, the calculated reductions are approximately 52.87%, 53.33%, 53.76%, 54.12%, and 54.41%, respectively. For example, the fitted forces at 0.10 m/s are 0.7996 cosθ and 0.37684 cosθ N; at 0.30 m/s, they are 1.7996 cosθ and 0.82036 cosθ N. These values are arithmetic evaluations of the existing fits, not new CFD outputs. The full expressions do not support a uniform 56% reduction. The retained summary does not specify the paired force data or averaging definition needed to reconcile that historical percentage. Interpretation is limited to the fitted-model comparison and the numerical verification boundary in Section 3.3.

R_arm_(v) = 1 − (0.3214 + 5.544v^2^)/(0.6746 + 12.5v^2^). (8)

### 4.4. Simulation Comparison Before and After Optimization

**Figure 9:**
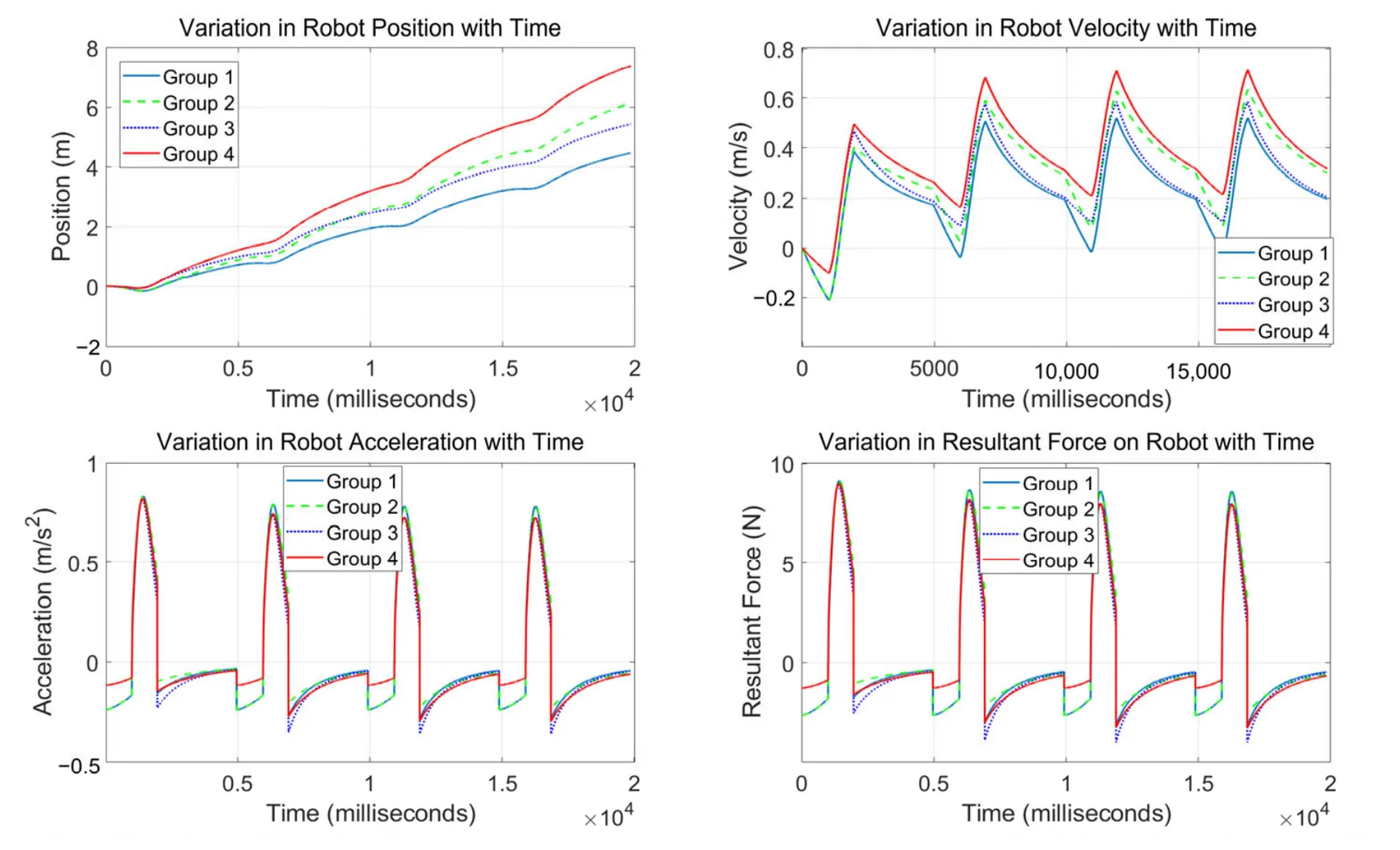
MATLAB comparison of four structural configurations within the same motor–spring actuation architecture; the comparison does not isolate motor-only versus motor–spring actuation.

Four configurations were compared in MATLAB, as shown in Figure 9: the original structure, body streamlining alone, arm modification alone, and the combined structural modifications. The configurations used the same actuation architecture and the corresponding fitted hydrodynamic relations. The retained simulated curves show higher velocity and smaller velocity fluctuations for the combined configuration relative to the original structure. These are model-based configuration comparisons; experimentally improved stability is not established by these curves.

This comparison does not isolate motor-only actuation from motor–spring actuation. A controlled motor-only comparison and measured energy consumption results are not available in the retained records. The configuration results therefore do not establish an independent spring contribution, a quantitatively demonstrated actuation synergy, or an energy efficiency advantage.

### 4.5. Streamlined Body Optimization and Variation in Total Drag

**Figure 10:**
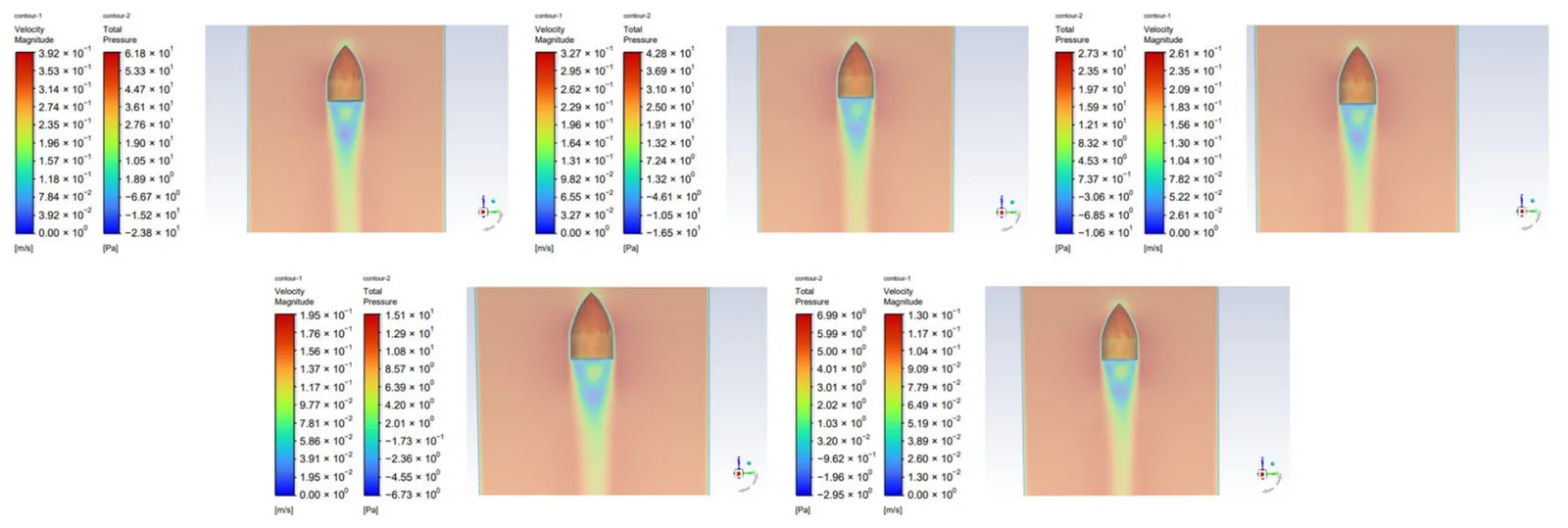
Reported body flow-field comparison before and after streamlining. The body-drag fits are interpreted separately from the recovery-stroke arm fits.

The retained body comparison used inlet velocities of 0.10, 0.15, 0.20, 0.25, and 0.30 m/s. The reported contours describe the original compartment and the streamlined body with a fairing, while Equations (9) and (10) give the corresponding fitted drag relations [18], as shown in Figure 10. These body results are considered separately from recovery-stroke arm drag.

F_D,original_ = 11.62v^2^. (9)

F_D,streamlined_ = 6.45v^2^. (10)

At a common nonzero inlet velocity, Equation (11) gives approximately 44.49% lower drag for the streamlined body fit. This arithmetic difference does not reproduce the historical 45.5% value or an exact 50% reduction, and the underlying averaging records do not resolve those values. Because a common reference area is not documented, the ratio is not reported as a measured or independently established change in drag coefficient. The body and arm percentages are neither added nor interpreted as a verified whole-robot drag reduction.

R_body_ = 1 − 6.45/11.62 ≈ 44.49%. (11)

## 5. Experimental Results

### 5.1. Straight-Line Swimming Experiment and Comparison with Simulation

The recorded straight-swimming summary gives approximately 1.8 m travel over approximately 9 s, corresponding to 1.8 m/9 s = 0.20 m/s, or approximately 20 cm/s overall. The same source reports approximately 10 cm/s during recovery and approximately 27 cm/s averaged during the combined power-stroke and gliding phases [18]. The 27 cm/s value is not treated as a measured maximum swimming speed. The historical 20.5 cm/s summary has no recoverable unrounded displacement, timing, or averaging record; it is not used as the principal overall speed and is not attributed to rounding. Table 2 uses the distance-to-time summary consistently.

**Figure 11:**
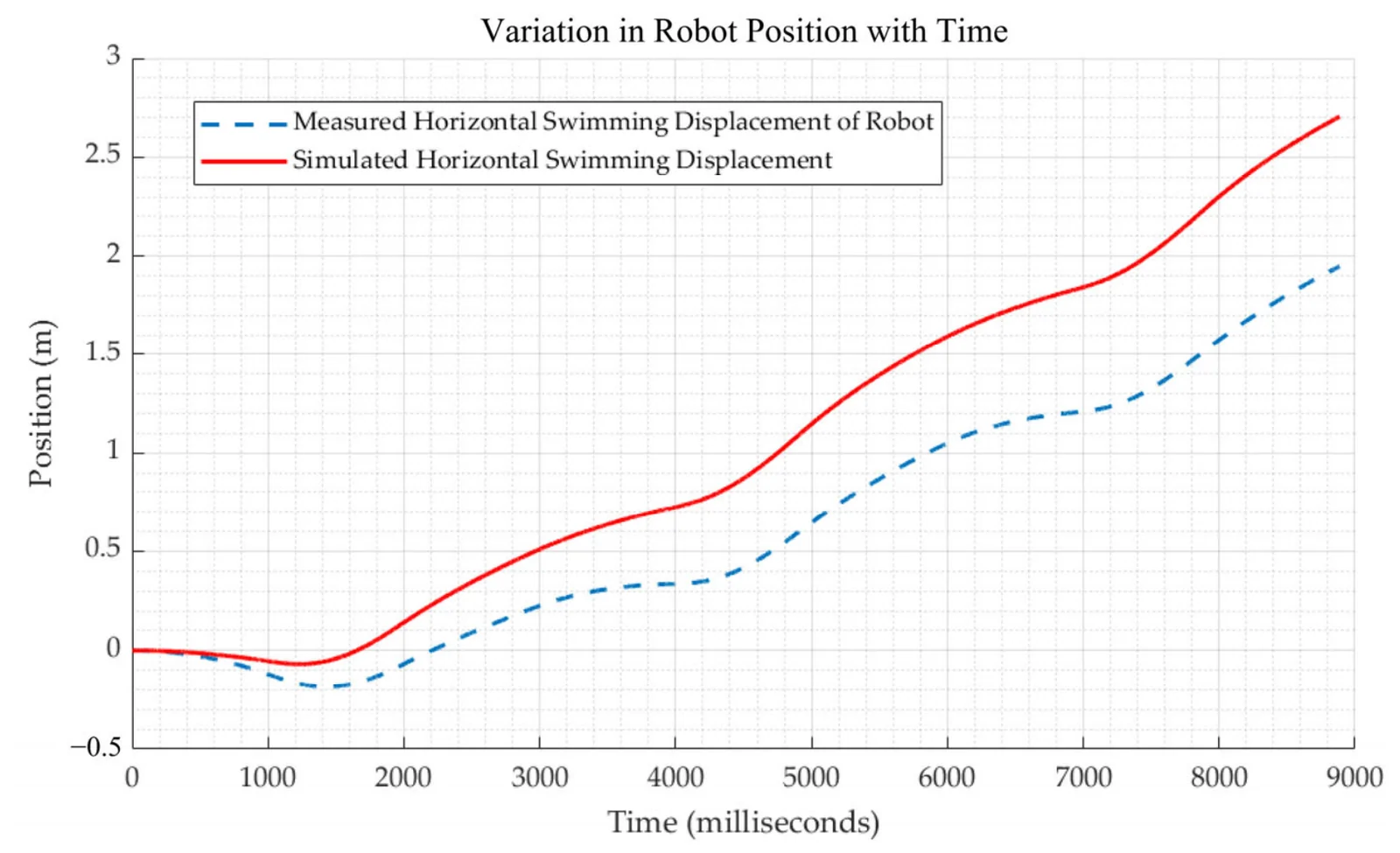
Retained illustrative displacement overlay used for qualitative interpretation. The documented tank protocol is approximately 3 s per cycle, and the separate MATLAB motion-cycle protocol is approximately 5 s per cycle; the overlay generation and time alignment procedure are not recoverable. Quantitative summaries follow the source text, rather than values digitized from this image.

The retained experimental and MATLAB overlay in Figure 11 shows qualitative phase-dependent trends: slowing during recovery, acceleration during the power stroke, and velocity decay during gliding. The documented timing protocols differ, and the overlay does not specify how its time axes were aligned. Together with the absence of matched raw time-series records, this prevents a defensible RMSE or percentage error calculation. The comparison is not quantitative model validation. The source reports surface-floating operation and occasional emergence of one arm; tank boundaries and resistance from the actual structure are also possible influences, but their individual contributions have not been quantified.

The recorded swimming sequence shows forward propulsion over consecutive cycles, supporting the feasibility of operating the integrated prototype with the prescribed actuation sequence. This observation does not quantify independent trial repeatability or isolate the effect of either the arm design or spring-assisted actuation.

### 5.2. Turning Experiment and Maneuverability

Turning was obtained by keeping the arm surface on the turning side closed while the other three were opened during the power stroke. The retained experimental summary reports a maximum turning angular velocity of approximately 4.2°/s [18]. This observation demonstrates a turning function under the reported tank conditions; its independent trial variability and measurement uncertainty are not established.

## 6. Discussion

### 6.1. From Morphological Imitation to Functional Translation

The biological inspiration is translated into three coordinated functions: motor-driven spring charging and release, direction-dependent arm response, and recovery–power-stroke–gliding timing. The contribution is the integration of these functions into one robot architecture. The present study evaluates this architecture through component hydrodynamic models, whole-robot motion analysis, and prototype observations, without assigning an isolated performance gain to each mechanism.

The numerical comparisons suggest lower fitted resistance for the modified arm and body geometries under the recorded model assumptions. Tank observations show straight swimming and turning of the integrated prototype. These two evidence paths support an engineering interpretation of the design but are not interchangeable: swimming observations do not measure drag reduction, and geometric CFD does not establish mechanical stiffness. The four-configuration study also does not provide motor-only versus motor–spring ablation.

Table 3 provides descriptive performance context for representative jellyfish-inspired robots. Values are limited to the literature summaries retained in the dissertation [18] and the present prototype records. The cited systems differ in geometry, actuation, test conditions, and speed definitions; the table is not a controlled ranking. No BL/s or cost of transport is calculated to fill missing records.

### 6.2. Functional Boundaries of the Asymmetric-Stiffness Design

The directional response arm combines hinged segments, return micro-springs, and a limiting structure to permit bending in recovery and resist bending in the power-stroke direction. Its response depends on geometry, local spring properties, membrane behavior, and fluid loading. Measured stiffness, moment–angle, and load–deformation data are not available in the retained records. The term asymmetric stiffness therefore describes the design intent; the magnitude of the asymmetry and its separate contribution to propulsion have not been quantitatively verified.

### 6.3. Limitations and Future Work

The conclusions are limited to the documented design, preliminary numerical comparisons, and recorded near-surface tank observations. Mesh independence and complete solver reproducibility are not established from the retained records. The residual plot cannot replace force convergence verification. Camera/calibration details and independent-trial statistics are insufficient to quantify measurement uncertainty or repeatability. The imported arm geometry does not supply coupled FSI deformation, and quantitative mechanical characterization and controlled actuation efficiency comparisons are not available. The differing MATLAB and tank timing limits their comparison to qualitative trends. Future work should address these specific verification needs and examine fully submerged operation, feedback control, and energy consumption under controlled conditions.

## 7. Conclusions

This paper presents a biomimetic jellyfish robot that integrates asymmetric-stiffness swimming arms and dual-power cooperative actuation. The main conclusions are as follows.

A motor–spring cooperative actuation architecture and an integrated prototype were implemented to organize recovery, power-stroke, and gliding phases. The head body accommodates the actuation, power supply, controller, buoyancy adjustment, and center of gravity adjustment components.

The swimming-arm concept combines hinged rigid segments, return micro-springs, and a directional limiting structure. It is designed for recovery-stroke bending and power-stroke shape retention; quantitative stiffness asymmetry is not established experimentally.

The retained CFD fits give preliminary arm geometry and body geometry comparisons. Arithmetic evaluation gives approximately 52.87–54.41% arm-drag reduction over 0.10–0.30 m/s and a separate approximately 44.49% body-drag reduction. These fitted-model differences are not direct experimental drag reductions, complete-robot drag reductions, or mesh-independent predictions.

The near-surface tank summaries record approximately 1.8 m travel over approximately 9 s, an overall average straight-line speed of approximately 20 cm/s, approximately 27 cm/s averaged during the combined power-stroke and gliding phases, and a maximum turning angular velocity of approximately 4.2°/s.

Together, the design, numerical comparisons, and prototype observations support the engineering feasibility of the mechanism–structure–function integration. Simulation–experiment interpretation is qualitative. The results do not establish quantitative stiffness validation, independent trial repeatability, or an isolated actuation efficiency advantage.

## Figures and Tables

**Table 1:** Main physical parameters of the prototype.

Parameter	Value
Total mass of the prototype	10 kg
Overall dimensions	740 mm × 270 mm × 270 mm
Body dimensions, diameter × length	270 mm × 390 mm
Number of swimming arms	4
Length of a single swimming arm	330 mm
Power supply	24 V, 6000 mAh lithium battery, independent power supply
Main controller	Microcontroller; model not confirmed (Section 2.5)
Swimming-arm servomotors	4; opening and closing of the swimming-arm surfaces
Motors	3; energy storage, center of gravity adjustment, and buoyancy adjustment
Sensor communication	IIC interface for the depth and attitude sensors
Wireless communication	ATK-LORA-01, 433 MHz
Main motor position measurement	Optical encoder
Power disconnection devices	Relays and an external physical power switch
Actuation mode	Motor energy storage–spring energy release
Working stroke of the main transmission rod	100 mm
Main parameters of the power spring	Wire diameter: 2 mm; outer diameter: 40 mm; free length: 300 mm
Working parameters of the power spring	Preload: 21 N; maximum compression: 100 mm; maximum output force: 70 N

**Table 2:** Recorded motion performance summaries under the near-surface tank protocol.

Performance Indicator	Experimental Result
Total duration of three consecutive motion cycles	Approximately 9 s
Average travel distance over three consecutive motion cycles	Approximately 1.8 m
Single-cycle duration	Approximately 3 s
Average speed during the slow recovery stroke	Approximately 10 cm/s
Average speed during the rapid power-stroke and gliding phases	Approximately 27 cm/s
Overall average straight-line speed	Approximately 20 cm/s
Maximum turning angular velocity	Approximately 4.2°/s

Note: The overall speed is calculated from the reported approximately 1.8 m/9 s. The 27 cm/s entry is a combined power-stroke-and-gliding-phase average, not a peak speed. The source mentions multiple repetitions but does not specify the independent trial count or individual trial data; no mean ± standard deviation is inferred from the three consecutive cycles.

**Table 3:** Comparison using the retained literature summaries [18]. Mean and maximum speeds, turning radius and angular velocity, and power and COT are reported separately.

(**a**) Physical parameters and swimming speed.						
**System/Reference**	**Actuation**	**Dimensions (cm)**	**Mass (kg)**	**Mean Speed (cm/s)**	**Maximum Speed (cm/s)**	**BL/s**
Cyro [6]	DC motor/linkage	Diameter 170	76	8.47	NR	NR
Najem IPMC [5]	IPMC	NR	NR	0.15	NR	NR
Magnetic miniature [19]	Magnetic	Height 3.5; width 1.5	NR	NR	NR	0.73 (max)
Wang [11]	Motor/linkage	Diameter 87.5 open; 44.5 closed; height 30	NR	NR	12	NR
Chu SMA [13]	SMA	Diameter 21; height 8	NR	NR	5.28	NR
LM-Jelly [15]	Electromagnetic/liquid metal	Diameter ≈ 9	NR	0.6	NR	NR
Present robot	Motor–spring	74 × 27 × 27	10	≈20	NR	NR
(**b**) Turning and energy-related information.						
**System**	**Minimum Turning Radius (cm)**	**Maximum Turning Angular Velocity (°/s)**		**Power Consumption (W)**		**COT**
Cyro	NR	NR		NR		NR
Najem IPMC	NR	NR		1.14		NR
Magnetic miniature	NR	NR		NR		NR
Wang	NR	NR		NR		NR
Chu SMA	31.95	NR		NR		NR
LM-Jelly	NR	NR		NR		NR
Present robot	NR	≈4.2		NR		NR

Notes: NR means unavailable in the retained records, not zero and not necessarily absent from the original publication. Dimensions retain their source definitions. Speeds are unit-converted from the reported summaries; no normalized speed is derived. The magnetic miniature swimmer’s 0.73 BL/s is a maximum normalized speed explicitly reported in the retained summary, whose citation corresponds to [19]. The present robot also has a recorded average speed of approximately 27 cm/s during the combined power-stroke and gliding phases, which is not included in the maximum speed column. Its values refer to the near-surface tank protocol. Voltage, battery capacity, and actuation frequency are not energy consumption measurements. The literature entries reproduce the retained summaries and are not independently verified primary study data.

## Data Availability

The original contributions presented in this study are included in the article. Further inquiries can be directed to the corresponding author.
